# A qualitative study of the perspectives of health workers and policy makers on external support provided to low-level private health facilities in a Ugandan rural district, in management of childhood infections

**DOI:** 10.1080/16549716.2021.1961398

**Published:** 2021-09-06

**Authors:** Juliet Mwanga-Amumpaire, Joan N. Kalyango, Karin Källander, Radhika Sundararajan, Judith Owokuhaisa, Joseph Rujumba, Celestino Obua, Tobias Alfvén, Grace Ndeezi

**Affiliations:** a Department of Paediatrics and Child Health, Mbarara University of Science and Technology, Mbarara, Uganda; b Clinical Epidemiology Unit, College of Health Sciences, Makerere University Kampala, Uganda; c Department of Pharmacy, College of Health Sciences, Makerere University Kampala, Uganda; d Department of Global Public Health, Karolinska Institutet, Sweden; e Center for Global Health, Weill Cornell Medicine, New York, USA; f Department of Microbiology, Mbarara University of Science and Technology, Mbarara, Uganda; g Department of Pediatrics and Child Health, College of Health Sciences, Makerere University Kampala, Uganda; h Department of Emergency Medicine, Sachs’ Children and Youth Hospital, Stockholm, Sweden

**Keywords:** Primary-level private facilities, health-care, support, childhood infections

## Abstract

**Background:**

With the under-five child mortality rate of 46.4 deaths per 1000 live births, Uganda should accelerate measures to reduce child deaths to achieve the Sustainable Development Goal 3. While 60–70% of frontline health services are provided by the private sector, many low-level private health facilities (LLPHF) are unregistered, unregulated, and often miss innovative and quality improvement strategies rolled out by the Ministry of Health. LLPHF need support in order to provide quality health care.

**Objective:**

To explore the perspectives of health workers and policy makers on external support given to LLPHF providing health care for children in Mbarara District, Uganda.

**Methods:**

We carried out a qualitative study, in which 43 purposively selected health workers and policy makers were interviewed. The issues discussed included their views on the quantity, quality, factors determining support received and preferred modalities of support to LLPHF. We used thematic analysis, employing an inductive approach to code interview transcripts and to identify subthemes and themes.

**Results:**

The support currently provided to LLPHF to manage childhood illnesses is inadequate. Health providers emphasised a need for technical capacity building, provision of policies, guidelines and critical supplies as well as adopting a more supportive supervisory approach instead of the current supervision model characterised by policing, fault finding and apportioning blame. Registration of the health facilities and regular submission of reports as well as multi-stakeholder involvement are potential strategies to improve external support.

**Conclusion:**

The current support received by LLPHF is inadequate in quantity and quality. Capacity building with emphasis on training, provision of critical guidelines and supplies as well as and supportive supervision are key strategies for delivering appropriate external support to LLPHF.

## Background

In low-income countries of Sub-Saharan Africa, one child out of 13 dies before their 5th birthday, compared to one out of 199 in high-income countries and 1out 264 in New Zealand and Australia[1,2]. Uganda has an under-five child mortality rate of 46.4 deaths per 1000 live births[3]. Between 60% and 70% of the deaths among the under-fives are due to treatable infectious causes namely malaria, pneumonia, neonatal infections and diarrhoeal diseases [4,5]. Significant progress in diagnosing and treating paediatric infectious diseases must be made to achieve the Sustainable Development Goal (SDG) 3 by reducing under-five mortality to 25/1000 live births by 2030.

Uganda’s Ministry of Health (MOH) recommended numerous child-survival strategies in order to reduce under-five mortality. These strategies included use of long-lasting insecticide treated mosquito nets to prevent malaria, immunisation, and management of common childhood illnesses [4]. However, poor uptake and implementation of these interventions at health facilities are important barriers in achieving reduction in childhood mortality [6].

In addition to e public health facilities private health facilities play an important role in health care provision in Uganda. In fact 60–70% of frontline health services and care for half of febrile children are provided by private primary-level health care facilities [4]. Prior research has described several factors driving utilisation of private health care facilities, including medication stock outs, long waiting time, health worker negative attitudes, non-availability of healthcare workers, and distant public health facilities [4,7,8].

While the private health sector are expected to participate in delivering the national minimum healthcare package (NMHCP) [9], most strategies for management of childhood illness rolled out by the MOH have mainly targeted public institutions. The private health facilities, especially at the lower level are often left out and end up functioning independently of the national health support systems [10,11]. This has led to some private health facilities often violating medical standards of practice, resulting in poorer patient outcomes [12]. Some exaggerate incentives from unnecessary testing and treatment, often have poor infrastructure, lack basic diagnostic instruments, and employ health workers with poor clinical competence [12–14]. Sub-optimal quality of care from these facilities may contribute to sustained child morbidity and mortality from easily treatable diseases. Provision of external support such as supportive supervision, clinical guidelines, and drug subsidies especially to low-level private health facilities (LLPHF) where the need is more pronounced [15] should improve the quality of health care for children at such facilities. While there is a draft on national policy of public private partnerships in health (NPPPPH), it does not spell out clearly the technical assistance given to PFP health facilities especially the LLPHF [16]. In fact supportive supervision seems to target mainly public and PNFP health facilities. There is paucity of information on support supervision received and needed by LLPHFs in relation to child health services. The aim of this study was therefore to generate evidence for strengthening support to LLPHF as a strategy to improve child health services. We specifically explored the perspectives of health workers in LLPHF as well as policy makers, regarding external support provided to these facilities in the management of common childhood infections. External support is referred to any form of assistance, be it technical, financial or otherwise, received by the private clinics from MOH and the District Health Teams (DHT) or other MOH affiliated bodies such as National Drug Authority (NDA), health professional regulatory bodies or non-government organisations (NGOs).

## Methods

### Research design and setting

This was a qualitative study nested in a large quantitative survey of quality of health care for common paediatric infections in 110 LLPHF selected randomly. The results of the survey are not included in this manuscript. The qualitative design was used to enable an in-depth exploration and understanding of the participants’ own experiences and perspectives regarding support received by LLPHF providing child health services in Mbarara District. The study was carried out among three stakeholder groups with different roles: (1) health care providers in LLPHF, (2) and policy makers in Mbarara District comprising the District Health Management Team, (3) and policy makers at the MoH in Uganda. The MoH is responsible for planning and formulating national health policies, and, through the professional bodies, regulation of health service providers. The MoH also supervises the district health office (DHO). Under the leadership of the District Health Officer, the DHO plans, organises, and oversees the implementation of the national policies by the different stakeholders in the district and supervises all healthcare facilities, except regional referral hospitals which are directly supervised by the MoH. The DHO performs its duties through the district health management team (DHMT) consisting of managers of different departments of health in the district, and heads of health centre IV which form the health sub-districts (HSD). The health facilities are the frontline providers of the health services to the public and should follow the MoH policies [17]. While the DHO does the direct supervision of both public and private health facilities in their jurisdiction, the healthcare workers are regulated by their different professional bodies; the Uganda Medical and dental Practitioners Council (UMDPC) for medical doctors, the Uganda Nurses and Midwives Council (UNMC) for nurses and midwives, and, the Allied Health Professionals Council (AHPC) for clinical officers [16].

**Figure 1.**
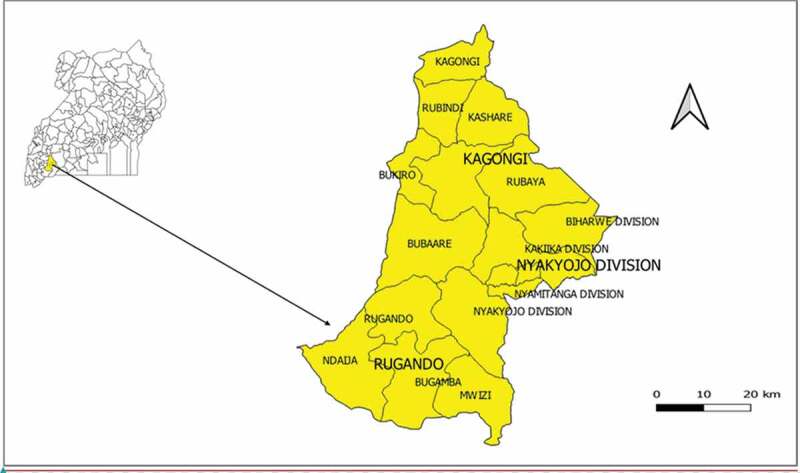
Map of Uganda showing location of Mbarara District. Extended is the map of Mbarara district, showing the sub counties where the study was carried out

Mbarara is located 267 km south-west of Kampala, Uganda and includes 3 administrative counties, 16 sub-counties, 83 parishes and 742 villages. Figure 1 is the map of Uganda showing the location of Mbarara District.

The district is primarily rural with a population of about 470,000 inhabitants and a density of 99 inhabitants/km. The main health facility is Mbarara Regional Referral Hospital, a tertiary referral centre that also serves as a teaching hospital for the Mbarara University medical school. The health facilities in the district follow the national MOH facility classification [18] defined by the population, catchment area served and the services offered as illustrated in Table 1.

**Table 1.** The health facility classification in Uganda

Level of Health Unit	Target population	Services provided and structures
Village Health Teams (Health Centre I)	1,000.	First contact for populations living in rural areas providing community-based preventive and health- promotion services, community mobilisation and referral of sick members to health facilities. No physical structures
Health Centre II	5,000	Parish-level facility offering Disease Prevention, Health Promotion and Outpatient Curative Health Services for uncomplicated conditions, antenatal care and immunisation for children.
Health Centre III	20,000	Sub county-level facility offering Preventive, Health Promotion, Outpatient Curative, Maternity, inpatient Health Services and Laboratory services for malaria testing and tuberculosis microscopy
Health Centre IV	100,000	County-level facility offering disease Preventive services, Health Promotion, Outpatient Curative, Maternity, inpatient Health Services, Emergency surgery and Blood transfusion and Laboratory services
General Hospital	500,000	District-level facility. In addition to services offered at HC IV, offers general services and in-service training, consultation and research to community based health care programs.
Regional Referral Hospital	2,000,000	In addition to services offered at the general hospital, offers specialist services, such as psychiatry, Ear, Nose and Throat, Ophthalmology, dentistry, intensive care, radiology, pathology, higher-level surgical and medical services.
National Referral Hospital	10,000,000	Offers comprehensive specialist services and are involved in teaching and research.

Source: Ministry of Health Annual Health Sector Performance report 2018–2019.

One hundred and twenty-four private facilities were registered with the national regulatory authorities in 2017 and are uniformly distributed throughout the District. Majority of the private health facilities are at HCIII level and lower. For the purposes of this study, we defined such facilities as low level private health facilities (LLPHF). These facilities have minimal infrastructure and treat common paediatric diseases such as uncomplicated malaria, pneumonia and diarrhoea and may provide immunisation. A few have small in-patient services limited to a maternity ward and a laboratory for simple diagnostics like malarial blood slides and urine tests. Many private clinics are run by nurses, midwives and clinical officers whose highest level of qualification are certificates or diplomas, while a few are headed by medical officers with medical degrees. In more rural facilities, the services may even be delivered illegally by individuals with minimal or no prior health-related training [15,16].

### Sampling and recruitment

Purposive sampling of health care providers in LLPHF and policy makers was used to select key informants with rich experience pertinent to the study topic. The health care providers were identified by the facility heads from 30 randomly selected LLPHF that offered child health services and had been in operation for at least 2 years. Ten health facilities were picked from each county and only one health care provider was interviewed from each of these LLPHF. This enabled capturing the heterogeneity among the health care providers and allowed for inclusion from rural and urban settings. Inclusion criteria for the providers were: (1) providing clinical care for children in private facility for at least 6 months; and (2) being employed at a LLPHF. Healthcare provider participants included nursing assistants, nurses/midwives, clinical officers and medical doctors. We also recruited 13 policy makers, in cooperation with their supervisors using the following inclusion criteria: (1) employed at their post for at least 2 years; and (2) directly involved with ensuring quality of child health in the district or MOH headquarters. Policy maker participants included a quality assurance officer from the MOH, 12 officials from the DHT including seven from the health sub-districts (HSD). A DHT is the district-level health service delivery decision-making body and is composed of the district health officer as the head, the heads of different departments at the district health office (DHO) and heads of the HSD. A health sub-district is the lowest Ministry of Health administrative structure and directly supervise the lower public health facilities and are in turn supervised by the DHO. All individuals who were approached, except one, accepted to participate in the study. One policy maker accepted to be interviewed but failed to find an appropriate time because of a busy schedule. The sample size of 43 participants was determined through data saturation, or the point when additional interviews did not provide novel insight or point to new concepts [19,20]. All participants provided written informed consent, including permission to audio-record the interviews. The study was explained to the participants in detail and they were given opportunity to ask questions and to withdraw from the study at any time if they did not wish to continue with the interview. Participants were informed that the data would be published and some of their quotes could be reported verbatim but their names would not be mentioned. After completion of the interview, participants received 20,000 Uganda Shillings (~$5 US dollars) as compensation for participation, the standard for research studies in the country.

### Data collection

Data collection consisted of a single qualitative interview. In-depth interviews were conducted between May and December 2019 by two female (BK and PT) and 1 male (CO) Ugandan research assistants (RAs) with prior training and experience in qualitative research. All interviewers were fluent in English and Runyankore, the dialect spoken by majority of people in Mbarara. The RAs did not know any of the study participants before conducting the interviews. Prior to study initiation, the RAs were trained for 3 days on the study protocol, principles of qualitative data collection and how to conduct high-quality interviews, interview translation and transcription. An interview guide was specifically created for this study to ensure consistent focus on the following topics among participants: (1) current support received by LLPHF (2) factors influencing support received by the LLPHF; (3) preferred modalities of support; and (4) suggestions to improve support to LLPHF. The interview guide questions were developed using input from Mbarara DHT officials, regarding topics of discussion pertinent to improving paediatric care at LLPHF. The interview guide was piloted at three private facilities, but the responses were not included in the analysis. The interviews were conducted in a private room at the respondents’ respective work places at a time and in their preferred language. Only the RA and the respondent were present in the room. Each interview lasted approximately 60 minutes and were audio-recorded with participant’s permission. Most of the interviews were carried out in the English language as per participant request (N = 40). All interviews were transcribed verbatim by the interviewer, based on audio recordings. For the three interviews conducted in Runyankore, concurrent transcription and translation was done into English. All transcripts were proof read by the first author, who speaks both languages fluently, for quality and translational integrity. The first author read the transcripts line by line within 48 hours of transcript completion and provided feedback to the RAs to continuously improve their interview skills throughout the data collection period. This ensured consistency in quality and content across all interviews, and served as a means to monitor for data saturation. To ensure anonymity the participants and their health facilities were identified by numbers, not names. In case of data from the district offices and ministry of health headquarters, where it was not possible to disguise the location, anonymity was assured by use of pseudonyms to de-identify the respondents and dropping the gender of the respondents. To ensure confidentiality, transcripts and the voice recordings were transferred to a password protected locked computer only accessed by the first author, who then shared password protected transcripts with the co-author involved in the coding of the data. Printed documents were kept in a locked cupboard accessible only to the first author.

### Analytical process

Interview data were analysed using thematic analysis [21]. The interviews were considered iteratively in order to facilitate exploration of additional themes and to actively monitor for data saturation. Two of the authors reviewed each transcripts several times, and following a combined deductive/inductive process [22] independently developed an initial set of codes. The two authors then discussed and compared their codebooks. Through consensus the codes were revised to create a final code list and subthemes, which were grouped together and fitted under the themes relevent to the research question. A preliminary list of subthemes and themes were shared and discussed with the authors and one independent peer who is a paediatrician with experience in qualitative research. Through further discussion final themes and corresponding subthemes in relation to perceptions regarding external support received by private health care providers were developed and are presented here with illustrative quotes from interview transcripts. The research team was made up of healthcare workers, researchers and academicians with different expertise, nationalities and backgrounds. Six of the authors are Ugandans, two are Swedish and one is American. Three are paediatricians, one is a molecular biologist, one a pharmacist, one a social scientist, one nurse and a microbiologist, one is a physician and one is a pharmacologist. We reflected on the impact of our backgrounds on the different phases of the research process. ATLAS.ti (GmbH, Berlin) was used for data organisation [23].

## Results

Most (n = 39, 90.7%) of the participants had been in their post for more than 1 year. Their characteristics are shown in Table 2.

**Table 2.** Characteristics of the study participants

Characteristic	Private health care workers N = 30, n (%)	Policy makers N = 13, n (%)
**Gender**		
Female	12(40)	6 (46)
Male	18 (60)	7 (54)
**Duration in service**		
6 months to 1 year	4 (13)	0 (0)
>1 to 5 years	12(40)	3 (23)
>5 years	14 (47)	10 (77)
**Profession**		
Nursing assistant	3(10)	0(0)
Nurse/Midwife	17(57)	5(39)
Clinical Officer	6 (20)	3(23)
Medical doctor	3(10)	2(15)
Other*	1(3)	3(23)

*Other – 1 laboratory assistant, 1 health educator, 2 health inspectors.

Our data are described under four themes pertinent to the support provided for LLPHF: (1) importance of external support, (2) nature of current support received by LLPHF, (3) support preferred by the LLPHF, and, (4) suggestions to improve support to LLPHF.

Table 3 shows the summary of these themes, their corresponding categories and sub-categories.

**Table 3.** Summary of the themes and their categories and sub-categories

Sub-categories	Categories	Themes
Make medical care affordable Mitigate providers’ knowledge gaps	Improve access and quality of health care provided by LLPHF	Importance of external support to LLPHF
Guidelines Vaccines Inspection	Types of support received by the LLPHF	Nature of current support received by LLPHF
Guidelines are rare in lower private facilities, Supervision is more fault finding than supportive	Appraisal of the current support received by LLPHF	
Training Support supervision	Technical capacity building	Support preferred by the LLPHF
Guidelines Supplies Subsidies	Tools and supplies	
Registration of LLPHF Regular submission of reports Join existing bureaus Commitment by LLPHF	Self-initiative of the LLPHF to comply with regulations	Suggestions to improve support to LLPHF
Ministry of health District NGOs Clinic owners	Multi-stakeholder involvement	

### Importance of external support to LLPHF

External support to LLPHF was stated as necessary to improve access and quality of health care provided by (1) making medical care for children affordable, and (2) mitigating providers’ knowledge gaps. For some patients the user medical costs are prohibitively expensive and deter caretakers from seeking care for their unwell children from private health facilities. Healthcare workers at LLPHF believed that external support could enable clinics offer care at a lower cost, thereby increasing utilisation of the health services by paediatric patients.

When someone comes (to the clinic) and the bill is beyond one hundred thousand Shillings ((˷USD 26) and yet has less than that, the next day he will not come back when the child falls sick. But if clinics are helped so that immediate care was offered at a cheaper cost or even at a free cost in some private centre … it will help a lot. (**Male medical doctor, urban clinic)**

Policy makers raised concern that some LLPHF healthcare workers have inadequate knowledge to treat childhood conditions and limited knowledge of standard treatment guidelines and need training to improve quality of care they offer.

Most health workers in private sector rarely receive trainings so which means there is a knowledge gap … .the care they offer may not be in line with the standards of the ministry of health. They need support to improve their knowledge (**HSD official)**

### Nature of current external support received by LLPHF

The current external support received by the LLPHF is described in two categories (1) types of support received, and, (2) appraisal of the current support received.

#### Types of support received

Study participants mentioned that some LLPHF received treatment guidelines and registers while a few facilities are supplied with vaccines and fridges to support the ministry of health in immunisation activities.

We receive some support from the district; vaccines, some booklets like the Uganda clinical guidelines and sometimes registers for recording laboratory tests and the patients we see. (**Male medical doctor urban clinic**)

It was noted that private clinics are often inspected by regulatory authorities with particular focus on the status of license and staffing. In some instances health workers mentioned receiving technical advice during such visits as one of them explained:

Inspectors come to look at their license, they want to know if you are using qualified people and if they have some time may check on the hygiene of the facility (**Female nurse rural clinic)**

#### Appraisal of the current support received

Participants at all levels acknowledged that even though some external support is being extended to LLPHF, it is inadequate. New guidelines are rarely disseminated to lower private facilities except in a few areas where such is supported by implementing partners, who are government or non-government entities that supplement the ministry of health in carrying out health-related projects.

The national level developments a lot of policies, standards and guidelines. We have very good guiding documents but the actual dissemination and their use at lower level private facilities is not well monitored. We have not really supported implementation. We have left it to partners so where you find UNICEF, Save the Children you find some work but still it’s not good enough. **(MoH official)**

Most study participants mentioned that supervision of private clinics was infrequent and when it was carried out, it was more of policing and fault finding than supportive. It was noted that MOH and DHO officials mainly visit clinics to check for licensure and stolen government drugs, but rarely to offer technical support. A common practice was to close clinics that do not comply and this keeps LLPHF healthcare providers apprehensive about interacting with those who may otherwise provide technical support.

Currently they are so much interested to know whether we have the government things around. They don’t want to see if we are segregating the waste well they only want to see if we have Panadol (Paracetamol) that has government of Uganda mark. (**Male clinical officer, urban clinic)**

NDA closes some of those clinics and the Uganda Medical and Dental Practitioners’ Council also looks for registration as well and closes those that do not have proper registration. There has been a lot of policing these days by medical associations. **(MOH official)**

Study participants also noted that most capacity building opportunities and support such as health worker training and mentorship targeted health workers at public facilities and not LLPHF;

When training is conducted, they only focus on government facilities and the private sector is always abandoned, if the private sector can also be brought on board it will bring a very big impact. (**Male nurse rural clinic)**

When the opportunity comes, the health ministry and other partners will only sponsor and support the government headed facilities so most of the time it becomes very difficult to train and update the private health workers. They have not had enough support … (**District official**)

### Support preferred by and for the LLPHF

Study participants described the nature of support preferred to strengthen the role of LLPHF in the provision of child health services as (1) technical capacity building, and, (2) provision of tools and supplies.

#### Technical capacity building

Study participants emphasised a need to train health workers on evidence-based strategies and the guidelines such as the Integrated Management of Childhood Illnesses (IMCI) as an important support for continuous skills improvement in care of common paediatric infections. Most participants felt that supervisory visits should be more supportive in nature.

Government should put up management standards in these facilities and give the guidelines. But you find some people cannot access the guidelines … they are working 10 years and never been supervised … The IMCI guidelines are not hard, if we can roll them out in as many facilities and even lower cadres the better. And still of course training people not to look at the money only but look at the holistic care of the children. **(Male doctor, urban clinic)**

#### Provision of tools and supplies

Provision of materials such as rapid diagnostic tests, medicines and subsidising operation costs of private clinics was another preferred form of support by health workers at LLPHF to enable them provide affordable lifesaving first line of care especially to patients who cannot afford:

The government should provide some medicines at no cost or subsided price to make private health services affordable by all so when someone comes we treat and give drugs even if they don’t have money. They need quality treatment to save the child’s life but they do not have money sometimes. **(Male nurse, rural clinic)**

### Suggestions to improve support to LLPHF

Study participants described two ways in which the external support could be improved; (1) self-initiative of LLPHF to seek help and comply with regulations, (2) multi-stakeholder involvement.

#### Self-initiative of the LLPHF to seek help and comply with regulations

Most participants noted that LLPHF need to bring their clinics to the attention of health authorities by seeking their help, registering with regulatory authorities, submitting regular reports to the DHT and MOH and in addition get affiliated to the existing bodies such as the religious medical bureaus through which funding to private health facilities is channelled by the government.

We have to actively seek help. What we should always do is to reach out to government. I go to them on a day of vaccination and ask for vaccination materials. They give me a vaccine batch but I take back the balance after completing the day. **(Medical doctor, urban clinic)**

The health worker at a private clinic at the lower level need to have their own initiative to first of all be registered with the district so that they are known so they can be supported by the district. Anything that is rolled out at the national level usually go through the district … . They should be reporting; the report is very important if you are not reporting you don’t benefit from government programs. They can help in the roll out of government programs such as giving free services like ARVs (anti-retroviral therapy) and early infant HIV diagnosis. However, the biggest problem is, many of them don’t have the capacity to do so … And these days the funding has been restricted to facilities affiliated to religious medical boards or bureaus; such as Uganda Protestant or Uganda Catholic or Uganda Moslem Medical Bureaus. They are no longer giving any private sector funding directly unless they are affiliated to any of those. (**Official from MOH)**

Policy-maker participants also underscored the importance of planning jointly with LLPHF but also stressed that LLPHFs should show commitment to take part in capacity building activities and in reducing staff turnover to guarantee sustainability of the capacity building efforts.

We can improve the participation of the clinic owners by getting them on board and planning together. If the clinic director is well trained and has the guidelines he can always pass it on to the health workers. The clinic owners should commit to train and keep their staff and if there is a way ministry or regulatory bodies would streamline the turnover of staff in private as this has been a gap. Even when we train this person you go back for follow up and they are no longer there. **(District official)**

#### Multi-stakeholder involvement

While all study participants recognised the mandate of the MOH to provide support to health facilities, they noted that everybody including facility owners and other partners in the health sector have a responsibility. Study participants preferred that the MOH oversees the supervision of private health facilities, sets the standard of care, gives guidelines and enforces regulation while the DHO and professional councils should offer support and supervision. It is preferred that NGOs offer other non-supervisory support. Regarding the need for multi-stakeholder engagement in improving support to LLPHF one district official explained;

We need to work as a team, all the stakeholders need to be brought on board, the district, partners, implementing partners, donors, so that we find a way forward. (**District official)**

## Discussion

In this study, we have documented views of health workers at LLPHF, district and national officials regarding the external support for LLPHF which provide child health care services. Findings reveal that external support is important in making child health services provided by the LLPHF affordable and of better quality. Previous research in Uganda have described improvement in appropriateness of care for febrile children when private drug shops were provided with subsidised drugs and diagnostic kits, training and supervision [24]. Often, rural populations may not afford fees charged for services provided by private clinics. Subsidising these costs by the government could improve uptake of services at these LLPHF and prevent delays in seeking care which has been described as a central factor contributing to under-five mortality [25]. Subsidising medical costs was piloted in selected districts in northern, eastern and western Uganda and lead to increased access to quality obstetrics and new-born services [26]. Increased health service utilisation was reported in Uganda when government contributed funding to PNFP health care facilities [10]

The current support received by LLPHF was in general considered inadequate in coverage and quality. It was noted that most LLPHF are excluded from health workers’ training and funding opportunities and often miss out on mentorship and important communication from the MOH including on changes in policies and guidelines. This is contrary to what is indicated in the national public—private partnership policy where even private health practitioners should benefit from in-service training [16]. Exclusion of health workers at LLPHF from training and mentorship support constitutes a missed opportunity to improve the already weak human resource capacity at these health facilities. In lowresource settings, rural health facilities often have underqualified healthcare cadres [11,13] who need professional support through training in standards for paediatric care. When supervision is perceived as policing and fault finding than supportive, as was the case in our study, the LLPHF health workers evade the supervisory visits. Harassment and arrests of health workers by regulatory authorities has been reported by other researchers in Uganda [27] and yet as demonstrated by Hill et al supervision strategy with a supportive approach gives better results than policing [28]. The current model of supervision to LLPHF should be therefore be adjusted to reflect the supportive role expected from supervisors for better quality improvement outcomes. Even in public facilities where support supervision has been happening, it has not been as comprehensive and therefore not had the full impact. It is for this reason that the MoH recently revised the national support supervision guidelines [29,30]. Like in other LMICs, the previous model of supervision by the Ugandan MoH employed top-down approach and utilisation of external specialists and was further weakened by limited resources and lack of coordination especially at the district level [30–33] This lack of resources was also a finding in our study. Supportive models of supervision which involve joint problem identification and solving by supervisor and mentee have shown better effects in other African countries [34,35,36].

Findings also revealed a preference for support in the form of clinical guidelines, rapid diagnostic tests, thermometers, weighing scales and subsidising costs of running the private facilities. This in part reflects current inadequacies in the availability of basic items needed to provide quality health care to children by LLPHF. Lack of basic materials in small private health facilities has previously been described in central Uganda [37] as a major capacity gap in the care for under-five children with febrile illnesses. Studies carried out in LMICs including Uganda have demonstrated improvement in quality of care when such support is given to lower level facilities [27,38–41]. A systematic review of studies carried out in sub-Saharan Africa demonstrated a need for financial and human resource input in order to achieve a sustained improvement in quality of healthcare [35]. This, however, may not be sustainable for an already resource constrained health sector. Alternative modes of financing from the government and donors, such as loan schemes, tax incentives on targeted items, contractual arrangements and purchase of medical services through vouchers or result-based funding could help offset the costs faced by the rural poor as was done for maternal and reproductive health [16,42].

Our findings revealed that in order to receive better support, the LLPHF need to commit and fulfil the conditions qualifying them for support such as registration, regular reporting of facility activities to health authorities and getting affiliated to already existing medical bureaus. However, it is important for health authorities to communicate and find innovative and easier ways for the LLPHF to register with regulatory bodies and bureaus. Often the small private facilities do not get to know about such opportunities, a phenomenon that has been described by other research in LMICs [42–44]. A multi-stakeholder approach to provision of support to LLPHF was recommended in our study given the varied needs of private health care providers. However, as the custodian of health, there is need for the ministry of health to take lead, to ensure that the support provided to the LLPHF is keeping with the MOH policies [16,29] and is better coordinated for maximum impact. Indeed, participants in this study suggested that the MOH maintains the regulation role but support supervision should be delegated to structures near the health facilities such as the health sub-districts. While this will bring services closer and ensure that support supervision is carried effectively it will only be functional after capacity building for mentors and supervisors at the district level as well as increasing resources to the district. This is in keeping with the recently revised national support supervision guidelines that mention formation of regional entities for this activity [29].

### Limitations and strength

One limitation is that we did not interview officials representing regulatory bodies and missed to capture their perspectives on support to LLPHF. The strength of this study lies in the fact that we employed qualitative methods and explored the perspectives of both health workers and policy makers on support rendered to LLPHF. We included a broad range of policy makers from highest to the lowest level of decision making and supervision as well as a variety of health workers.

### Conclusions

Our study has shown that the current support received by LLPHF is inadequate in quantity and quality. Supervision is more fault finding than supportive and only few clinics receive this supervision. Capacity building with emphasis on training and provision of critical tools including policies, guidelines and supplies as well as adapting a more supportive form of supervision with the involvement of stakeholders are key strategies for delivering appropriate external support to LLPHF.

At facility level, to improve access to the needed support, clinic owners should comply with operational guidelines, register with the relevant professional councils and submit regular performance reports as required by the MoH. The DHO and MoH, should support LLPHF to form coalitions that they identify with easily through which support to the LLPHF can be channelled. To ensure sustainable support the DHO should regulate the number of facilities to a manageable level, and support only those facilities that register annually. This will in the long run, weed out the illegal facilities that provide poor-quality services as clients will chose facilities offering quality services. In keeping with the World Health Assembly resolution 69.11 of strengthening health systems with emphasis on the poor and vulnerable populations [45],to ensure equity in delivery of quality healthcare to the rural poor there is need to support these primary level private health facilities. In order to achieve the universal health coverage (UHC), the MoH together with international bodies such as UNICEF and WHO, need to support not only public facilities or high-level private health facilities. Such support can be done by identifying and putting in place innovative training and mentoring strategies, ensuring availability of guidelines and ensuring that national and international policies reach these calibres of healthcare providers.

## Panel summarising the recommendations

**Table:** 

Facility levelMandatory registration of private clinics with regulatory bodies Submission of performance reports to the MoH and DHO regularly Joining already existing organisations such as medical bureaus to access support District health office levelAdapting a more supportive form of supervision with the involvement of stakeholders Regulate the quantity of private clinics in the district to manageable numbers Identify and encourage formation of LLPHF coalitions through which support can be delivered Ministry of health and national and international development partnersIdentify and encourage formation of LLPHF coalitions through which support can be delivered. Identifying and put in place innovative training and mentoring strategies Ensure availability of guidelines and that national and international policies reach these calibers of healthcare providers

## Paper context

Half of the care for febrile children in Uganda are provided by low-level private clinics yet these receive inadequate support to guarantee quality health care. We explored the views of health workers in low-level private clinics and policy makers on how they could be supported to deliver quality health care. Technical capacity building remain feasible strategy of delivering support to low-level private clinics but the clinics need to register with regulatory bodies and report regularly.
